# Analytical Performance Evaluation of the New GEM^®^ Premier™ 5000 in Comparison to the Epoc^®^ Blood Gas Analyzer in Horses

**DOI:** 10.3390/vetsci10020114

**Published:** 2023-02-03

**Authors:** Charlotte Sandersen, Petra Dmitrovic, Julien Dupont, Carla Cesarini, Hugues Guyot, Didier Serteyn, Katharina Kirsch

**Affiliations:** 1 Department of Clinical Sciences, Faculty of Veterinary Medicine, University of Liege, 4000 Liege, Belgium; 2 Department of Clinical Sciences, Faculty of Veterinary Medicine, University of Zagreb, 10000 Zagreb, Croatia; 3 German Olympic Committee for Equestrian Sports, 48231 Warendorf, Germany

**Keywords:** blood gas analysis, horse, Passing–Bablok analysis, Bland–Altman analysis, method comparison

## Abstract

**Simple Summary:**

Blood gas analyzers are stationary or hand-held machines used to measure blood gases and electrolytes in a blood sample taken from a patient. No machine has been specifically designed for use in horses, and therefore every machine needs to be validated before being used in equine practice. The aim of this study was to compare the newly marketed GEM5000 machine to the formerly validated epoc machine for blood gas analysis in horses. Blood samples taken from healthy and sick horses were run on both machines in alternate order, and various statistical tests were used to analyze if the two machines give similar results. Although the precision of the GEM5000 is good for most parameters, the agreement with the epoc machine is not always satisfactory. Therefore, data from different blood gas machines should not be used interchangeably.

**Abstract:**

Different blood gas analyzers are used in equine practice. Every machine needs to be validated, as they have not been designed for use in horses. The aim of this study was to compare the newly marketed GEM5000 machine to the formerly validated epoc machine for blood gas analysis in horses. In this prospective, non-blinded, comparative laboratory analyzer study, 43 equine blood samples were analyzed on both analyzers and values were compared between the two machines via Lin’s concordance analysis, Passing–Bablok regression analysis and Bland–Altman plots. Duplicate measurements were conducted on the GEM5000 machine to evaluate precision. The GEM5000 failed to achieve the required precision for tHb, Hct and iCa^2+^, but achieved acceptable precision for all other parameters. Concordance correlation analysis revealed poor correlation for Na^+^, Cl^−^, iCa^2+^, K^+^, Hct and tHb, while there was an at least moderate agreement for all other parameters. Passing–Bablok regression revealed significant constant bias for pCO_2_, pO_2_, Cl^−^, and iCa^2+^ and significant proportional bias for pCO_2_, iCa^2+^ and SO_2_. Bland–Altman analysis revealed significant systematic bias for Na^+^, Cl^−^, iCa^2+^, K^+^, Hct, tHb and SO_2_. This study shows that while precision of the GEM5000 is good, values should not be used interchangeably with data obtained from other blood gas analyzers.

## 1. Introduction

The blood gas and electrolyte analysis (BGEA) is an essential tool in equine medicine and research as it helps the veterinarian to establish a clinical diagnosis, a prognosis and to provide an appropriate therapeutic monitoring of patients. Most machines offer the possibility to measure pH, partial pressures of oxygen (pO_2_) and carbon dioxide (pCO_2_), as well as electrolytes such as sodium (Na^+^), potassium (K^+^), chloride (Cl^−^) and ionized calcium (iCa^2+^). 

BGEA is especially indicated in patients in whom acid–base or electrolyte imbalances are suspected [1,2,3], such as in critically ill patients, where BGEA helps in monitoring patients and guides fluid support according to their needs. Likewise, the use of BGEA has been recommended to monitor exercising horses, as high-intensity exercise is associated with alterations in acid–base and electrolyte balance [4,5,6,7,8,9,10,11,12]. In addition, arterial BGEA can be used to assess ventilation efficiency of the lungs [2], making it indicated for the monitoring of patients undergoing general anesthesia, patients suffering from cardiac or respiratory disease and critically ill foals [3]. 

Since BGEA has become common practice in equine medicine, in clinics as well as in field conditions, several analyzers, hand-held and stationary ones, have been used and validated for equine practice, although none of these analyzers have been specifically designed for use with equine blood. Therefore, each machine needs to be validated and results of different devices should not be used interchangeably without precaution [13]. Recent papers compared various machines [13,14,15], but no study has evaluated the GEM5000 analyzer in horses so far. 

The GEM5000 (Instrumentation Laboratory, Bedford, MA, USA) has been developed for the quantitative measurement of pH, pCO_2_, pO_2_, Na^+^, K^+^, Cl^−^, iCa^2+^, glucose, L-lactate, total hemoglobin (tHb), hematocrit (Hct), saturation of hemoglobin (SO_2_) and CO-oximetry parameters in human whole blood. This analyzer is equipped with a so-called iQM2 system that provides automated continuous quality assurance in real time. The iQM2 technology performs continuous checks, before, during and after each sample, while errors are automatically detected and corrected, claiming superior reliability of the machine. 

For the purpose of this study, the GEM5000 was compared to the epoc analyzer which has recently been validated for use with equine blood [13,14]. Our hypothesis was that the results obtained with the GEM5000 will not differ significantly from those obtained with the epoc machine, rendering the GEM5000 reliable for use in clinical settings for BGEA in horses.

## 2. Materials and Methods

### 2.1. Animals

Whole blood samples were collected from horses during their stay at the Equine Hospital of the University of Liege. Blood samples were taken from horses because of their primary clinical condition and only when deemed necessary by the clinician in charge. No extra blood was taken solely for the purpose of this study. In these conditions, no approval from the Ethical Committee of the institution was necessary. Owners of the horses agreed for the data of their horses and the results of the BGEA to be used for scientific purposes in an anonymized format. Forty-three blood samples from twenty-six horses admitted to the Equine Hospital of the University of Liege in January 2019 with various pathologies were used in this study. Horses with different diseases were recruited to ensure a wide range of blood gas partial pressures and electrolyte concentrations. 

### 2.2. Sample Collection

Blood samples were taken in prefilled heparinized syringes (Blood gas-Monovette^®^, Sarstedt AG & Co. KG, Nümbrecht, Germany) as a part of the clinical work-up of the patients or as a part of monitoring during general anesthesia. Blood samples were taken from the jugular vein or from the carotid artery in standing horses or from a catheter placed in the transverse facial artery in anaesthetized horses. 

### 2.3. Sample Analysis

Analyses were immediately performed after blood sample collection and both machines were used in alternating sequence. Both instruments were maintained, calibrated and operated according to the manufacturers’ guidelines. The devices were installed side by side to minimize the time interval between measurements. The following parameters were evaluated for the comparison of the two machines: pH, pCO_2_, pO_2_, Na^+^, Cl^−^, iCa^2+^, K^+^, Hct, tHb, BE, SO_2_ and HCO_3_^−^.

To evaluate the precision of the GEM5000 Premier machine, 29 samples were analyzed twice, in rapid succession. The following measured variables and calculated parameters were assessed for precision analysis: pH, pCO_2_, pO_2_, Na^+^, Cl^−^, iCa^2+^, K^+^, Hct, tHb, BE, SO_2_ and HCO_3_^−^.

### 2.4. Instruments

In both machines, epoc (epoc Blood Analysis System, Siemens Healthcare SA, Groot-Bijgaarden, Belgium), and GEM5000 (Gem Premier 5000, Zaventem, Belgium), the pH, pCO_2_ and electrolytes are measured potentiometrically with ion-selective electrodes and basing calculations on the Nernst-equation. Oxygen is measured with an ion-selective electrode of the Clarke type. The HCO_3_^−^ is calculated by using the Henderson–Hasselbalch equation in both instruments using the following equation (log [HCO_3_^−^] = pH + log pCO_2_ − 7.608). 

### 2.5. Statistical Analysis

Precision. To evaluate precision of the GEM5000 machine, samples were run in duplicate, and the mean, the standard deviation and the coefficient of variation (CV) in percent of each duplicate measurement were calculated for each parameter (Microsoft Excel). The means of all the CV were compared to preset clinical limits of acceptance with the assumption that they should be equal to or less than 25% of the defined allowable total error [16]. The allowable errors for blood gases and electrolyte concentrations were based on the Criteria for Acceptable Performance of the Clinical Laboratory Improvements Amendments [17]. 

Agreement. In order to determine the bias between the two analyzers, results obtained from the GEM5000 machine were compared to those obtained from the epoc machine using concordance correlation analysis, the Bland–Altman method, and the Passing–Bablok regression analysis (MedCalc, Ostende, Belgium). Lin’s concordance correlation coefficient (r_ccc_) has a measure for accuracy (bias corrected factor, Cb) and for precision (Pearson’s ρ). It serves as an indicator of the strength of concordance between two measurements [18]. A value of the r_ccc_ of less than 0.90 was considered as poor agreement, 0.90 to 0.95 as moderate agreement, 0.95 to 0.99 as substantial agreement and above 0.99 as almost perfect agreement between the methods [19]. 

The slope, the intercept and the residual standard deviation (RSD) of the Passing–Bablok regression analysis reflects the proportional, the constant bias and the random bias, respectively. If the 95% confidence intervals for intercept contained the value 0, and for slope the value 1, no significant constant or proportional bias between measurements is noticed. If data are normally distributed, 95% of random differences are expected to lie in the interval of −1.96 RSD to +1.96 RSD. A large interval is considered as an indicator for a high imprecision [20]. The linearity between compared results was assessed by the CUSUM test, which warrants the validity of Passing–Bablok procedure.

Additionally to regression analysis, Bland–Altman analysis determined the agreement between the compared methods by plotting the difference of two paired measurements against the mean of the two measurements. The mean difference between results of compared methods reflects the systematic bias. If the value 0 is included in the 95% confidence interval of the bias, it is assumed that there is no significant systematic bias [19,21]. Assuming that differences between measured values are normally distributed, 95% of all data points should lie within 1.96 times the standard deviation of the mean difference. This assumption allows for calculation of 95% limits of agreement [20,22]. The limits of agreement serve as an estimate of the total bias, which is composed of systematic and random bias and is assessed by the comparison with the pre-specified clinically allowable errors. Bias was assumed to be clinically relevant, if the 95% limits of agreement were outside the limits for the allowable total errors. The CLIA guidelines served as a basis for the assignment of limits for clinical acceptance [17]. 

## 3. Results

### 3.1. Animals and Samples 

A total of 43 blood samples including 23 arterial and 20 venous blood samples were collected and analyzed. These samples were taken from 26 horses of various breeds (19 Warmblood Horses, 2 Frisians, 2 Riding Ponies, 1 Draft horse, 1 Quarter Horse, 1 Standardbred) and ages. There were 3 stallions, 14 geldings and 9 mares, aged between 1 and 25 years. The mean body weight was 543 kg and ranged between 293 and 700 kg. 

### 3.2. Precision (Repeatability)

Table 1 displays the mean coefficient of variation (CV) of each of the 29 duplicate measurements obtained from the GEM5000 analyzer, with comparison to clinical limits of acceptance, which are defined as equal to or less than 25% of the defined allowable total error. 

### 3.3. Agreement

Table 2 shows the mean values, standard deviations and ranges for the measured parameters of the two machines. Table 3 shows the results of the concordance correlation analysis between the epoc and the GEM5000 analyzer. At least moderate agreement was obtained for pH, pCO_2_, pO_2_, HCO_3_^−^, BE and SO_2_, while the analysis revealed poor agreement for all measured electrolytes and tHb. 

**Figure 1:**
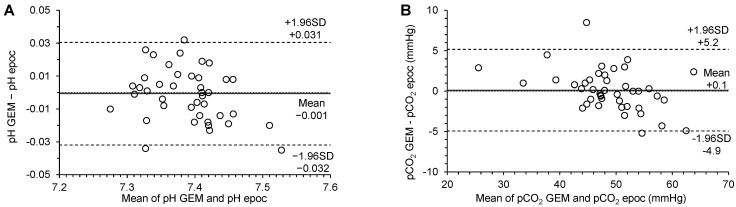

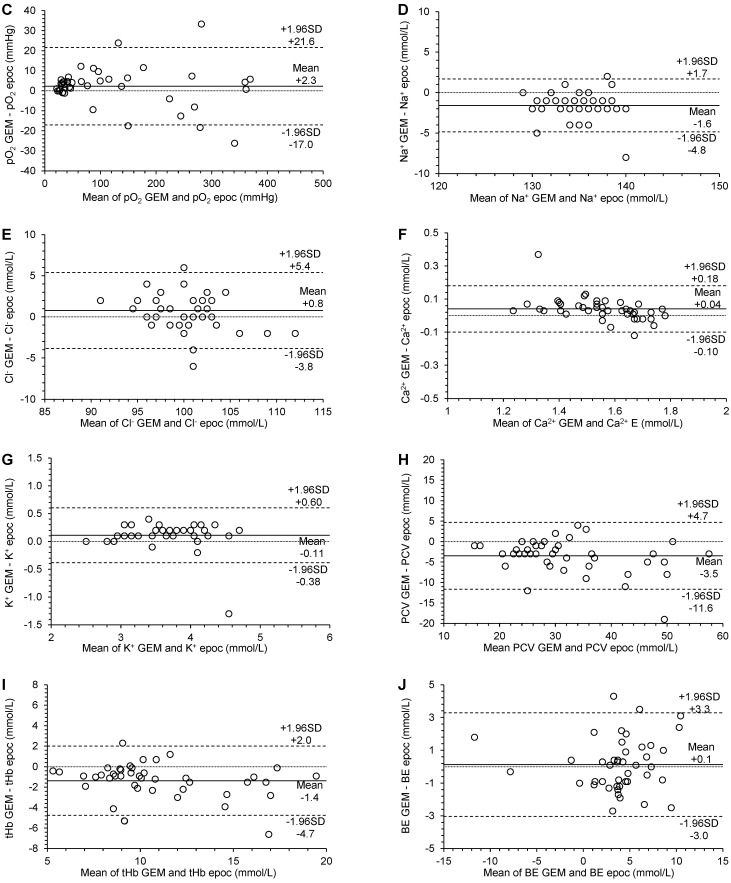

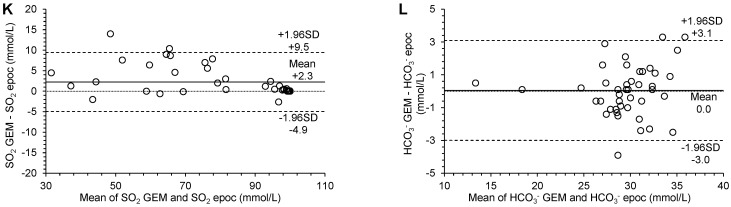
Bland-Altman plots for comparison of GEM5000 and epoc analyzers for blood pH (**A**), pCO_2_ (**B**), pO_2_ (**C**), Na^+^ (**D**), Cl^−^ (**E**), Ca^2+^ (**F**), K^+^ (**G**), packed cell volume PCV (**H**), tHb (**I**), BE (**J**), SO_2_ (**K**), [HCO_3_^−^] (**L**). The dashed lines are upper and lower limit of agreement, the solid line is the mean difference (bias).

Table 4 shows the results of the Passing–Bablok regression and the Bland–Altman analysis from the comparison between epoc and GEM5000 for all measured parameters. Figure 1 displays the Bland–Altman plots for the comparison between the two analyzers for pH (A), pCO_2_ (B) and pO_2_ (C), Na^+^ (D), Cl^−^ (E), iCa^2+^ (F), K^+^ (G) Hct (H), tHb (I), BE (J), SO_2_ (K) and HCO_3_^−^ (L). The CUSUM analysis revealed no significant deviation from the linear relationship between the compared results for all determined parameters (*p* > 0.05).

pH: For the pH, the Bland–Altman plot revealed no significant systematic bias between results obtained from the epoc and the GEM5000 (*p* = 0.7682). The Passing–Bablok regression revealed no significant constant or proportional bias.

pCO_2_: For the pCO_2_, the Bland–Altman plot revealed no significant systematic bias between results obtained from the epoc and the GEM5000 (*p* = 0.7512). The Passing–Bablok regression revealed a significant constant and proportional bias.

pO_2_: For the pO_2_, the Bland–Altman plot revealed no significant systematic bias between results obtained from the epoc and the GEM5000 (*p* = 0.1356). The Passing–Bablok regression revealed a significant constant but no significant proportional bias.

Na^+^, K^+^, Cl^−^, iCa^2+^: The Bland–Altman analysis indicated a significant systematic bias between results obtained from the epoc and the GEM5000 for Na^+^ (*p* < 0.000), K^+^ (*p* = 0.0056), Cl^−^ (*p* = 0.00364), and iCa^2+^ (*p* = 0.0006). The Passing–Bablok regression indicated no significant proportional bias for Na^+^, K^+^, Cl^−^, and no constant bias for Na^+^ and K^+^; however, there was a significant constant bias for Cl^−^. For iCa^2+^, the Passing–Bablok analysis revealed both significant constant and proportional bias. 

Hct: For the Hct, the Bland–Altman plot revealed a significant systematic bias between results obtained from the epoc and the GEM5000 (*p* < 0.0001). The Passing–Bablok regression revealed no significant constant or proportional bias.

tHb: For the tHb, the Bland–Altman plot revealed a significant systematic bias between results obtained from the epoc and the GEM5000 (*p* < 0.0001). The Passing–Bablok regression revealed no significant constant or proportional bias.

BE: For the BE, the Bland–Altman plot revealed no significant systematic bias between results obtained from the epoc and the GEM5000 (*p* = 0.6059). The Passing–Bablok regression revealed no significant constant or proportional bias.

SO_2_: For the SO_2_, the Bland–Altman plot revealed a significant systematic bias between results obtained from the epoc and the GEM5000 (*p* = 0.0002). The Passing–Bablok regression revealed no significant constant but a significant proportional bias.

HCO_3_^−^: For the HCO_3_^−^, the Bland–Altman plot revealed no significant systematic bias between results obtained from the epoc and the GEM5000 (*p* = 0.8377). The Passing–Bablok regression revealed no significant proportional or constant bias.

## 4. Discussion

Blood gases and electrolytes play a vital role in a number of disorders affecting the homeostasis of the patient. The delay to obtain laboratory results for iCa^2+^, Na^+^, K^+^ and Cl^−^ concentrations is often too long to allow for timely decision making in critically ill patients. Therefore, reliable methods for on-site measurements are critical. In addition, determination of blood gases usually requires an analysis immediately after blood sample collection, since storage and transportation to the laboratory would affect the results. Several studies have investigated the effect of syringe type and storage temperature on blood gas analysis and underline the importance of standardized storage conditions and minimal delay from sampling to analysis [24,25]. Different analyzers are used for this purpose, but no machine has been designed for specific use in horses. Therefore, every machine needs to be validated before use in clinics. The epoc analyzer has recently been tested for equine blood, but cannot be considered as a gold standard technique [13,14]. Precision targets were met by the epoc machine for pH, PO_2_, Na^+^, K^+^, glucose, lactate, HCO_3_^−^, BE (ecf) and SO_2_, but not for PCO_2_, iCa^2+,^ HCT and Hb [14], while agreement was poor for Hct, Hb and BE when compared to the iSTAT or an ABL machine. Kirsch and co-workers [12] reported that the epoc was not meeting precision targets for pO_2_, pCO_2_ and K^+^ and that agreement was poor for pH, [HCO_3_^−^] and [Na^+^] when compared with the Cobas analyzer. Despite these variable results for precision and agreement with other machines, the epoc was chosen as a comparison machine for the GEM5000 in the present study, as its precision was generally accepted as satisfactory. Actually, no blood gas analyzer can be considered as a gold standard, and when comparing two machines, it is not clear which one provides data that are closer to the real values. Apart from storage temperature and duration, the body temperature itself may affect the measurement. In the current paper, the alpha-stat method has been used and all measurements were run on the machines, assuming a body temperature of 37 °C. Although, the importance of body temperature and temperature correction for blood gas analyses has been previously highlighted in exercising horses [26], it is probably less relevant for a method comparison study. The alpha-stat hypothesis suggests that we always interpret our blood gases as corrected to the same temperature (normal body temperature), regardless of how high or low the body temperature actually is. The pH-stat hypothesis, on the other hand, suggests that temperature should always be corrected to the core body temperature. Each approach has its advantages and disadvantages and deciding to use either one is especially important when clinical decisions are based on the results [27]. Since the current study compares the analytical performance of two different blood gas machines, we assume that any temperature related error would be the same for the two analyzers.

Precision analysis of the GEM5000 revealed that results were acceptable for pH, pCO_2_ and for all electrolytes, while they were considered too high for Hct, tHb and iCa^2+^. When comparing to maximal CV% given by Westgaard [16], Hct and tHb were out of acceptable range. Hct and tHb are linked and a possible explanation is improper mixing. The importance of mixing the samples, especially for Hct, has been underlined by Mion and co-workers [28], who tested the GEM5000 machine at two different human hospitals. They measured the effect of different handling of the sample during the pre-analytical phase before analyzing the sample in a GEM500 machine. They compared the effect of (1) automatic mixing using a rotary shaker for 5 min only to (2) 5 min of automatic mixing followed by 30 s of gentle turning and inverting by hand and (3) 5 min of automatic mixing using a rotary shaker followed by quick inversions by hand for an additional 30 s prior to analysis. Their results showed that additional hand mixing is superior to machine only mixing and that the best result is obtained when flipping the tube rapidly for 30 s before analyzing. In the present study, all samples were handled by the same operator and no machine mixing was used. The tubes where gently inverted by hand for 30 s before analyzing. The time from sampling to analysis was kept short, but results of precision for Hct and tHb indicate that precision could have been improved by better mixing of the samples. Although efforts were made to standardize the conditions, such as minimizing the delay from sampling to analysis, mixing the sample, and working in controlled room temperature and pressure, some errors might have occurred in the pre-analysis period. Available evidence strongly suggests that the pre- and post- analytical steps are more error-prone than the intra-analytical phase [28].

As with most machines, the precision is good, while the agreement is poor. It can be suggested that data from a single machine are reliable, while comparing data from different machines should be done cautiously. This may be of relevance in clinical practice, where analyses are run on different machines.

One of the principal limitations of the present study is the low number of samples investigated. In general, it is recommended that 40 to 100 samples should be used for method comparison studies [29], but this also depends on the range of samples, which should be as wide as possible. More than half of the results, however, were outside the reference intervals for equine venous blood, indicating a wide range of data. This is recommended by the Clinical Laboratory Improvement Amendments (CLIA) guidelines for evaluating agreement between two analyzers [30]. The reference ranges used for comparison were not generated by the machines in question, and therefore the exact number of samples out of reference range cannot be determined. Future studies should focus on the establishment of reference values for healthy horses on the GEM5000 machine. The importance of species and equipment specific reference values has been underlined by a recent study, which established reference values for equine BGEA [31].

## 5. Conclusions

Considering the overall satisfactory analytical performance, the good practicability, the intuitive and easy to use software interface and the rapid measuring time (about 45 s from sample aspiration to results), GEM5000 seems suitable to be used in equine practice, but results obtained with this machine should not be used interchangeably with those from other blood gas analyzers.

## Figures and Tables

**Table 1:** Mean values and standard deviation, and coefficient of variation of the first sample as well as the mean CV for all 29 duplicate measurements of blood samples on the GEM5000 analyzer.

Measured Variables	GEM5000			
	Mean ± SD of the 1st Sample	CV (%) of the 1st Sample	Mean CV (%) of All Duplicates	Precision Target (CV%)
pH	7.53 ± 0.025	0.33	0.07	<0.1 [13,16]
pCO_2_ (mmHg)	38 ± 2	5.26	0.87	<2.4 [12,16]
pO_2_ (mmHg)	79.5 ± 8.5	10.69	3.18	<4.8 [12,16]
Na^+^ (mmol/L)	132 ± 0	0	0.3	<0.3 [12,16]
Cl^−^ (mmol/L)	95.5 ± 0.5	0.52	0.42	<0.6 [14,16]
iCa^2+^ (mmol/L)	1.67 ± 0.01	0.59	**1.1**	<0.9 [14,16]
K^+^ (mmol/L)	4.65 ± 0.15	3.22	0.8	<2.3 [14,16]
PCV (%)	31.5 ± 1.5	4.76	**3.84**	<1.35 [14,16]
tHb (g/dL)	10.7 ± 0.5	4.47	**4.84**	<1.43 [14,16]
BE (mmol/L)	8.8 ± 0.6	6.82	34.8	<38.2 [16]
SO_2_ (%)	96.75 ± 1.15	1.19	1.04	<5 [23]
HCO_3_^−^ (mmol/L)	32.05 ± 1.15	0.47	1.74	<5 [23]

pCO_2_, partial pressure of carbon dioxide; pO_2_, partial pressure of oxygen; Na^+^, sodium; Cl^−^, chloride; iCa^2+^, calcium; K^+^, potassium; Hct, hematocrit; tHb, total hemoglobin; BE, base excess; SO_2_, oxygen saturation; HCO_3_^−^, bicarbonate. Data over of precision target in bold.

**Table 2:** Mean, standard deviation (SD) and measured concentration range for parameters determined by the two compared analyzers epoc and GEM5000 (*n* = 43).

Measured Variables	epoc		GEM5000	
	Mean ± SD	Range	Mean ± SD	Range
pH	7.390 ± 0.056	7.280–7.545	7.390 ± 0.052	7.270–7.510
pCO_2_ (mmHg)	48.7 ± 7.7	24.1–64.9	48.9 ± 6.6	27.0–65.0
pO_2_ (mmHg)	117.9 ± 111.3	21.0–366.2	120.2 ± 109.7	22.0–372.0
Na^+^ (mmol/L)	136 ± 3	129–144	134 ± 3	128–139
Cl^−^ (mmol/L)	100 ± 4	90–113	101 ± 4	92–111
iCa^2+^ (mmol/L)	1.53 ± 0.16	1.14–1.78	1.57 ± 0.12	1.25–1.79
K^+^ (mmol/L)	3.6 ± 0.6	2.5–5.2	3.7 ± 0.5	2.5–4.8
Hct (%)	34 ± 11	16–59	30 ± 9	15–56
tHb (g/dL)	11.5 ± 3.7	5.5–20.2	10.2 ± 3.3	5.1–19.0
BE (mmol/L)	3.8 ± 4.1	−12.6–10.7	4.0 ± 4.1	−10.8–12.0
SO_2_ (%)	80.4 ± 22.0	29.3–100.0	82.7 ± 20.1	33.8–100.0
HCO_3_^−^ (mmol/L)	29.5 ± 3.9	13.1–35.8	29.5 ± 4.1	13.6–37.5

pCO_2_, partial pressure of carbon dioxide; pO_2_, partial pressure of oxygen; Na^+^, sodium; Cl^−^, chloride; iCa^2+^, calcium; K^+^, potassium; Hct; hematocrit; tHb, total hemoglobin; BE, base excess; SO_2_, oxygen saturation; HCO_3_^−^, bicarbonate.

**Table 3:** Concordance correlation analysis between the epoc and the GEM5000 analyzer, indicating the accuracy (bias correction factor, C_b_) and the precision (Pearson ρ) as well as the Lin’s concordance correlation coefficients (r_ccc_) with 95% confidence intervals (95% CI).

Measured Variables	Concordance Correlation Analysis			
	Lin’s r_ccc_ (95% CI)	Precision ρ	Accuracy C_b_	
pH	0.956 (0.923 to 0.976)	0.960	0.997	substantial agreement
pCO_2_ (mmHg)	0.935 (0.889 to 0.962)	0.946	0.989	moderate agreement
pO_2_ (mmHg)	0.996 (0.992 to 0.998)	0.996	1.000	almost perfect agreement
Na^+^ (mmol/L)	0.720 (0.569 to 0.824)	0.833	0.865	poor agreement
Cl^−^ (mmol/L)	0.811 (0.685 to 0.890)	0.839	0.967	poor agreement
iCa^2+^ (mmol/L)	0.837 (0.739 to 0.901)	0.897	0.933	poor agreement
K^+^ (mmol/L)	0.875 (0.785 to 0.929)	0.895	0.977	poor agreement
Hct (%)	0.865 (0.779 to 0.920)	0.925	0.936	poor agreement
tHb (g/dL)	0.814 (0.699 to 0.888)	0.885	0.921	poor agreement
BE (mmol/L)	0.922 (0.862 to 0.957)	0.923	0.999	moderate agreement
SO_2_ (%)	0.979 (0.965 to 0.988)	0.989	0.990	substantial agreement
HCO_3_^−^ (mmol/L)	0.926 (0.869 to 0.959)	0.927	0.999	moderate agreement

pCO_2_, partial pressure of carbon dioxide; pO_2_, partial pressure of oxygen; Na^+^, sodium; Cl^−^, chloride; iCa^2+^, calcium; K^+^, potassium; Hct; hematocrit; tHb, total hemoglobin; BE, base excess; SO_2_, oxygen saturation; HCO_3_^−^, bicarbonate.

**Table 4:** Results of the Passing–Bablok regression and the Bland–Altman plots resulting from the comparison between the GEM5000 and the epoc analyzer.

Measured Variables	Passing–Bablok Regression			Bland–Altman Plot						
	Intercept (95% CI)	Slope (95% CI)	Residual Standard Deviation (95% CI)	Bias (95% CI)	Lower Limit of Agreement (95% CI)	Upper Limit of Agreement (95% CI)	Passing Bablok		Bland–Altman	
pH	0.67 (−0.07 to 1.31)	0.91 (0.82 to 1.01)	0.011 (−0.022 to 0.022)	−0.001 (−0.006 to 0.004)	−0.032 (−0.040 to −0.024)	0.031 (0.022 to 0.039)	no significant constant bias	no significant proportional bias	no significant systematic bias	*p* = 0.7682
pCO_2_ (mmHg)	7.13 (2.38 to 11.30)	0.85 (0.76 to 0.95)	1.690 (−3.312 to 3.312)	−0.1 (−0.7 to 0.9)	−4.9 (−6.3 to −3.6)	5.2 (3.8 to 6.6)	significant constant bias	significant proportional bias	no significant systematic bias	*p* = 0.7512
pO_2_ (mmHg)	2.84 (0.24 to 5.00)	1.01 (0.98 to 1.04)	7.253 (−14.215 to 14.215)	2.3 (−0.7 to 5.3)	−17.0 (−22.2 to −11.8)	21.6 (16.4 to 26.8)	significant constant bias	no significant proportional bias	no significant systematic bias	*p* = 0.1356
Na^+^ (mmol/L)	−2.0 (−2.00 to 15.50)	1.0 (0.88 to 1.00)	1.230 (−2.410 to 2.410)	−1.6 (−2.1 to −1.1)	−4.8 (−5.7 to −4.0)	1.7 (0.8 to 2.6)	no significant constant bias	no significant proportional bias	significant systematic bias	*p* < 0.0001
Cl^−^ (mmol/L)	14.86 (1.00 to 27.82)	0.86 (0.73 to 1.00)	1.614 (−3.163 to 3.163)	0.8 (0.1 to 1.5)	−3.8 (−5.1 to −2.6)	5.4 (4.1 to 6.7)	significant constant bias	no significant proportional bias	significant systematic bias	*p* = 0.0364
Ca^2+^ (mmol/L)	0.34 (0.18 to 0.51)	0.81 (0.70 to 0.90)	0.045 (−0.088 to 0.088)	0.04 (0.02 to 0.06)	−0.10 (−0.14 to −0.06)	0.18 (0.14 to 0.22)	significant constant bias	significant proportional bias	significant systematic bias	*p* = 0.0006
K^+^ (mmol/L)	0.10 (−0.26 to 0.10)	1.00 (1.00 to 1.11)	0.180 (−0.352 to 0.352)	0.11 (0.03 to 0.19)	−0.38 (−0.51 to −0.25)	0.60 (0.47 to 0.74)	no significant constant bias	no significant proportional bias	significant systematic bias	*p* = 0.0056
HTC (%)	−0.47 (−3.00 to 2.90)	0.92 (0.81 to 1.00)	2.840 (−5.566 to 5.566)	−3.5 (−4.7 to −2.2)	−11.6 (−13.8 to −9.4)	4.7 (2.5 to 6.9)	no significant constant bias	no significant proportional bias	significant systematic bias	*p* < 0.0001
tHb (g/dL)	−0.04 (−1.23 to 1.12)	0.91 (0.79 to 1.02)	1.212 (−2.376 to 2.376)	−1.4 (−1.9 to −0.8)	−4.7 (−5.7 to −3.8)	2.0 (1.1 to 2.9)	no significant constant bias	no significant proportional bias	significant systematic bias	*p* < 0.0001
BE (mmol/L)	−0.10 (−1.45 to 0.34)	1.07 (0.91 to 1.33)	1.163 (−2.279 to 2.279)	0.1 (−0.4 to 0.6)	−3.0 (−3.9 to −2.2)	3.3 (2.4 to 4.1)	no significant constant bias	no significant proportional bias	no significant systematic bias	*p* = 0.6059
SO_2_ (%)	12.97 (6.10 to 15.73)	0.87 (0.84 to 0.94)	2.372 (−4.649 to 4.649)	2.3 (1.1 to 3.4)	−4.9 (−6.9 to −3.0)	9.4 (7.5 to 11.4)	no significant constant bias	significant proportional bias	significant systematic bias	*p* = 0.0002
HCO_3_^−^ (mmol/L)	−3.46 (−11.04 to 1.27)	1.12 (0.96 to 1.38)	1.116 (−2.188 to 2.188)	0.0 (−0.4 to 0.5)	−3.0 (−3.8 to −2.2)	3.1 (2.3 to 3.9)	no significant constant bias	no significant proportional bias	no significant systematic bias	*p* = 0.8377

pCO_2_, partial pressure of carbon dioxide; pO_2_, partial pressure of oxygen; Na^+^, sodium; Cl^−^, chloride; Ca^2+^, calcium; K^+^, potassium; HTC; packed cell volume; tHb, total hemoglobin; BE, base excess; SO_2_, oxygen saturation; HCO_3_^−^, bicarbonate.

## Data Availability

On publication of the article, data will be made available on an open science framework.
